# Novel Amorphous-Wollastonitic Low-Calcium Hydraulic Binders: A State-of-the-Art Review

**DOI:** 10.3390/ma16134874

**Published:** 2023-07-07

**Authors:** Mónica Antunes, Rodrigo L. Santos, Ricardo B. Horta, Rogério Colaço

**Affiliations:** 1 Instituto Superior Técnico, University of Lisbon, 1049-001 Lisbon, Portugal; monica.h.antunes@tecnico.ulisboa.pt (M.A.);; 2 IDMEC-Instituto de Engenharia Mecânica, University of Lisbon, 1049-001 Lisbon, Portugal; 3 CIMPOR—Cimentos de Portugal, SGPS S.A., 1099-020 Lisbon, Portugal

**Keywords:** amorphous-wollastonitic low-calcium hydraulic binders (AWLCs), clinker, alkaline activation, tobermorite, CO_2_ emissions

## Abstract

Because of the severe environmental impact of the CO_2_ emissions associated with the production of ordinary Portland cement (OPC) and the increasing demand for this commodity material, the development of alternative products has become a global concern. One alternative to OPC, or alitic-based clinkers, are amorphous-wollastonitic low-calcium hydraulic binders (AWLCs). This new class of hydraulic binders, described in the literature for the first time in 2015, may significantly reduce the CO_2_ emissions associated with its production, resulting from its lower calcium content, but also from the fact that its production technology can be fully electrified. In this paper, a state-of-the-art review is presented, providing a comprehensive description of the latest research, summarizing both the physicochemical and mechanical characteristics of this type of hydraulic binder, as well as possible routes for its production at an industrial scale.

## 1. Introduction

Ordinary Portland cement (OPC) is a hydraulic binder manufactured in a rotary kiln whose raw materials contain adequate amounts of lime, silica, and, in smaller proportions, alumina and iron oxide [1]. The mixture is first calcined at 900 °C, followed by a clinkering stage at 1450 °C to allow the formation of alite (CaO)_3_, SiO_2_, and, in smaller amounts, belite (CaO)_2_.SiO_2_ [1]. The produced clinker, with a density of ~3.1 g/cm^3^ [2], is then ground to an optimum particle size distribution [3], obtaining a specific surface area that ranges from 3000 to 5000 cm^2^/g [4,5,6]. When in contact with water, OPC goes through an exothermic hydration reaction responsible for the material’s strength development. This reaction can release more than 250 J/g of cumulative heat after 72 h of hydration [7].

OPC is mainly used in the production of mortar (cement mixed with water and sand) and concrete (cement mixed with water, sand, and other coarse aggregates). Concrete is extremely resilient and durable, can bear heavy compressive loads, and resist severe environmental conditions, making it the world’s most widely used construction material [7]. The main factors that influence the compressive strength of concrete are hydration time, type of cement used, and temperature and curing conditions [1]. OPC is not only a widely used material with well-established manufacturing technology but is also a low-cost commodity, as it is made from raw materials abundant in the Earth’s crust (SiO_2_ and CaO). Hence, because of these unique properties and the growing need for housing, services, and transport network development, cement consumption is expected to significantly increase in the next decade [8].

The use of OPC has a significant environmental impact due to the amount of CO_2_ released through its production, grinding, and distribution process [9]. The study in this work focused on the reduction of CO_2_ emissions associated with its production, which is the main contributor to the overall emissions [9].

In fact, for each ton of clinker produced, approximately 800 kg of CO_2_ is concomitantly produced, which means that the production of one ton of cement may result in the release of up to 540 kg of CO_2_ into the atmosphere [10,11]. These CO_2_ emissions result from two main sources: CO_2_ process emissions (CPEs) and CO_2_ energy emissions (CEEs). CPEs come from the chemical reaction involved in the decarbonation of limestone, while CEEs are related to the burning of fossil fuel required for heating the cement kiln [12].

Consequently, reducing the environmental impact of cement production has become an increasing concern. In the 2022 Energy Agency Report [8], the “near-zero emissions” concept is mentioned as a goal to achieve by cement industry by 2050, aiming for an over 80% reduction in emissions compared to the present best available technology.

Nevertheless, the quest for carbon neutrality depends on the rapid scale up in the deployment of alternative clinker technologies, such as alkali-activated materials (AAMs) [11] or low-calcium hydraulic binders (LCHBs), which may reduce CO_2_ emissions by decreasing the amount of CaCO_3_ used in their production [13,14,15,16,17,18,19,20].

LCHBs are materials rich in phases with lower calcium content than alite, such as belite, rankinite (CaO)_3_(SiO_2_)_2_, or wollastonite CaO.SiO_2_. Belite is a hydraulic phase but with slower hydration kinetics than alite because of its densely packed structure and reduced solubility, which lowers the compressive strength of the binder at early ages [21,22]. Both rankinite and wollastonite are nonhydraulically active; hence, binders rich in these phases usually go through a carbonation process instead of a hydration reaction [20,23].

In 2015, an alternative LCHB was developed and patented internationally [13,18]. The precursor idea involves the production of a material with a Ca/Si ratio of ~1, corresponding approximately to the chemical composition in which wollastonite crystallizes at equilibrium conditions [14] but processed in a way that promotes the formation of an amorphous phase upon cooling instead of the crystalline one. In fact, it was shown that by melting a mixture with a Ca/Si ratio of ~1 at a temperature between 1460 and 1550 °C, corresponding to the liquid or the liquid + pseudowollastonite zone, followed by a fast quenching, a material with hydraulic properties is obtained [13,14,18]. This material is mostly amorphous (~94% wt%), with the presence of a small proportion of pseudowollastonite (<10%). The reduction of the calcium content of this material results in a decrease of more than 25% of the CPE compared to alitic clinkers [11]. Moreover, the complete melting of these amorphous-wollastonitic low-calcium hydraulic binders (AWLCs) enables the full electrification of the production process [11]. Therefore, AWLC clinkers are prone to electrification because they do not need a rotary kiln, and there is complete melting of the material, which makes the overheating unproblematic [11]. Hence, theoretically, if electric energy derives from renewable green sources, the production of AWLCs has the potential to result in “near-zero” CEE emissions.

Several studies have been conducted to characterize the properties and hydraulic activity of AWLCs. The chemical composition and microstructure of AWLCs and their respective hydration products were determined using X-ray fluorescence (XRF), nuclear magnetic resonance (NMR), Fourier-transform infrared spectroscopy (FTIR), X-ray diffraction (XRD), and high-resolution transmission electron microscopy (HR-TEM) [14,24,25,26]. The hydration of the binder with water was characterized by measuring the compressive strength of pastes at various ages [18,24,27,28], and its chemical reaction was followed using isothermal calorimetry and computational work [29]. More recent works explored the impact of an alkaline activation of AWLC [16,30]. In the following, a state-of-the-art review concerning the production, characterization, and optimization of AWLCs, as well as perspectives for future developments, is presented.

## 2. Production of AWLC Hydraulic Binders

The first studies on AWLC binder focused on the influence of the following properties: Ca/Si ratio [15,26], melting temperature [15,25,26,27], and quenching method [24]. In all of these studies, the production process was carried out in small quantities, less than 450 g, and under laboratory conditions, following a three-step procedure [18]:

I. Heating the raw mixture at a rate R1 = 25 °C/min to reach the required melting temperature (T1);
II. Maintaining temperature (T1) in the liquid region for a period of t1 = 60 min to allow for the homogenization of the composition;
III. Cooling the system to room temperature.

**Figure 1:**
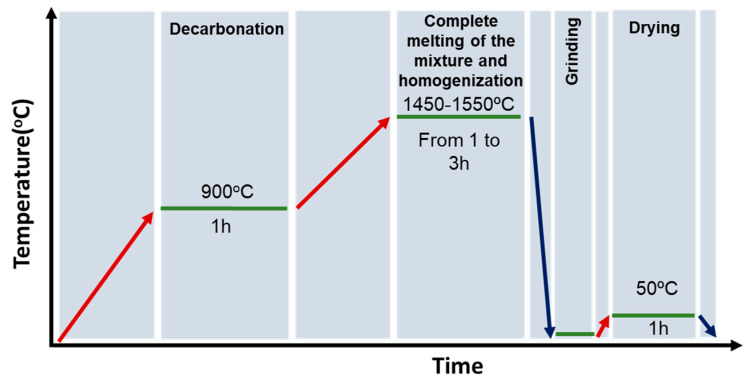
Schematic representation of the production process.

For the production of larger batches, an intermediate step between (I) and (II) was implemented by keeping the mixture at 900 °C for 1 h [30]. This implementation was required to allow for the efficient decarbonation of the mixture and to avoid gas accumulation in the batch [30]. After quenching, a material with a density of 2.94 ± 0.5 g/cm^3^ [26] was obtained. Finally, the obtained product was ground in a ring mill with propanol followed by a drying step at 50 °C for 1 h [30]. Figure 1 depicts a schematic representation of the production process.

## 3. Characterization of the AWLC

### 3.1. Anhydrous Material

**Figure 2:**
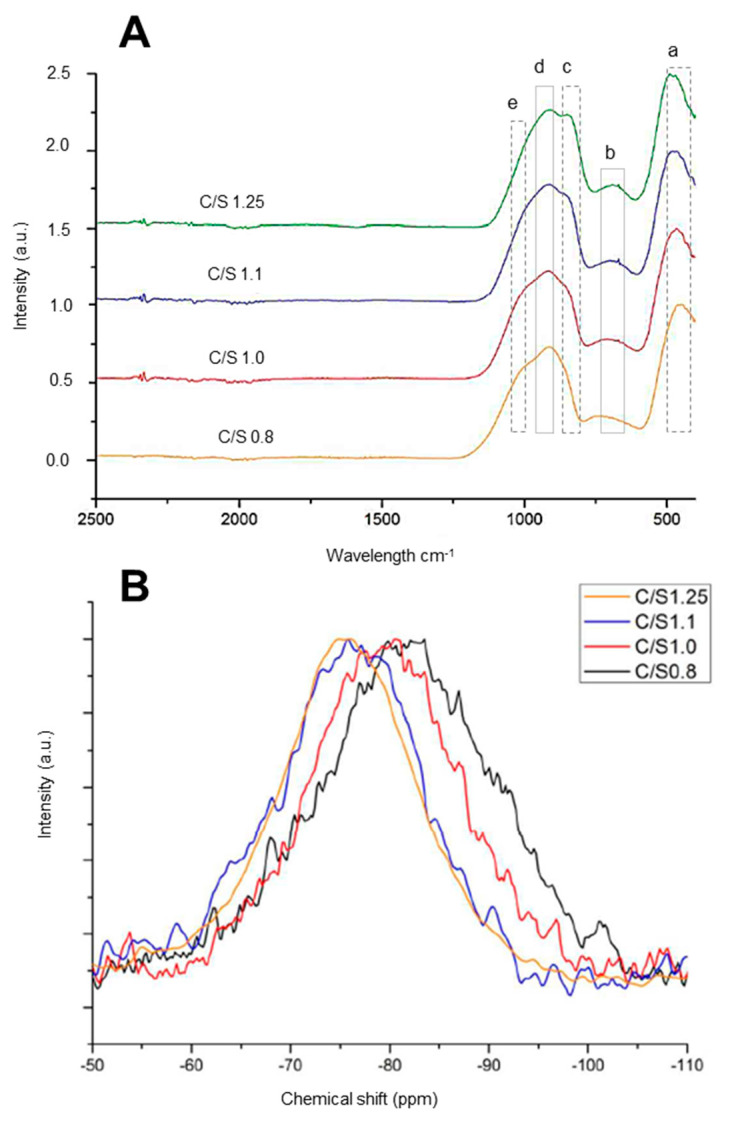
(**A**) FTIR spectra results of AWLC samples produced with a Ca/Si ratio in a range between 0.8 and 1.25; (**B**) ^29^Si MAS NMR spectra of AWLC samples produced with a Ca/Si ratio in a range between 0.8 and 1.25. Adapted from [26].

Figure 2 shows the FTIR and NMR analyses of samples of AWLC, with the Ca/Si ratios ranging from 0.8 to 1.25 (taken from [26]). An FTIR analysis of AWLC binders shows five main regions:

- 400 to 500 cm^−1^;
- 600 to 750 cm^−1^;
- 780 to 850 cm^−1^
- 870 to 900 cm^−1^;
- 900 to 1000 cm^−1^;
- 1100 to 1200 cm^−1^.

The peak identified as ***a***, located at approximately 450–490 cm^-1^, can be attributed to Si-O-Si bending [31] and was similar in all samples. The bands at ***b*** are caused by the tetrahedral Si-O-Si vibration of amorphous silica [32], and they increased as the Ca/Si ratio increased, indicating the presence of Q^1^ and Q^0^ structures in the sample [18]. The peak at ***c*** can be attributed to an isolated tetrahedra’s nonbridging Si-O stretching mode [33]. The bands ***d*** and ***e*** are related to the stretching vibrations of nonbridging Si-O bonds, indicating the presence of Q^2^ and Q^3^ units [18,32]. The shoulder at ***e*** was more prominent in samples with a lower Ca/Si ratio, indicating the formation of a more structured sample.

The deconvolutions spectra and results of the ^29^Si MAS NMR are shown in Figure 2, and Table 1, respectively. It can be observed that contrarily to what occurs with wollastonite, which is essentially formed by Q^2^ structures [33], AWLC is formed by the dispersion of Q^n^ connectivity, with a prevalence of Q^1^ units with a dispersion of Q^0^, Q^2^, and Q^3^. It can be observed that as the Ca/Si increased from 0.8 to 1.1, there was an increase in the proportion of Q^0^ units, and then the Q^0^ proportion decreased again. Hence, with the increase in the Ca content from 0.8 to 1.1, there was increasing depolymerization of the silicate chains [26].

### 3.2. Hydrated Material

#### 3.2.1. Isothermal Calorimetry

Since understanding the kinetics of the hydration reaction is crucial for evaluating the binder’s compressive strength, isothermal calorimetry measurements were used to track the hydration evolution of the material.

**Figure 3:**
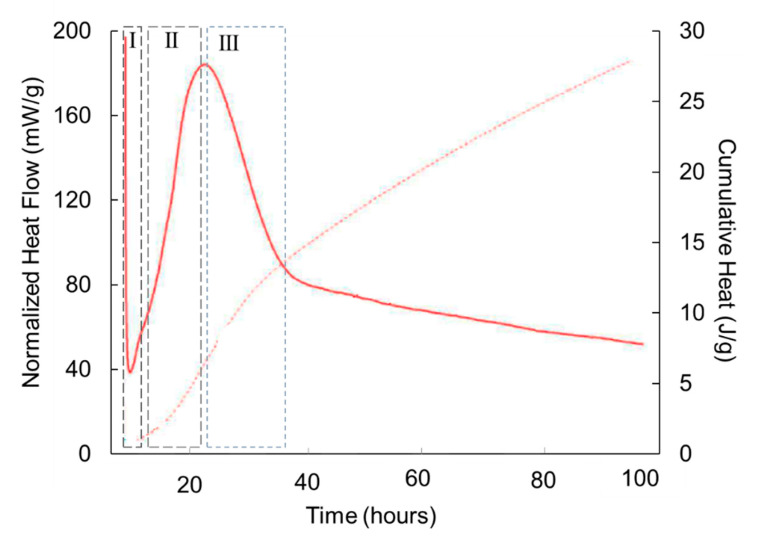
Normalized heat flow (solid) and normalized cumulative heat (dotted) curves as a function of time of hydration of an AWLC paste with a 0.325 water/binder ratio and a specific surface area of 5135 cm^2^/g, adapted from [24]. The calorimetric curve was divided into 3 sections: (**I**) initial period; (**II**) acceleration period; (**III**) deceleration period.

Figure 3 illustrates the three regions that can be observed in AWLC calorimetric experiments:

I. A first stage of the hydration, characterized by slow reaction kinetics, also observed in OPC, is usually attributed to species ionic dissolution [34];
II. An acceleration period characterized by a high rate of heat release [35,36]. In OPC, this stage is usually attributed to the precipitation of CSH products and portlandite. Since in AWLC there is no precipitation of portlandite, this second stage should correspond to the formation of CSH and, most probably, to the formation of tobermorite, which is present in the hydrated product [17], as we will see in the following points;
III. A deceleration period in which the rate of the reaction decreases, probably due to the inability of CSH to keep precipitating on the surface of the grains. In this period, a gradual densification of the microstructure occurs, as described for OPC [36,37].

The activation energy (E_a_) of AWLC pastes was recently calculated using the Arrhenius equation and calorimetric results in a range of temperatures from 20 to 35 °C [17]. The results showed that the AWLC had an experimental E_a_ in the 82–85 kJ/mol range [17], which is almost 50% higher than the E_a_ of alitic and belitic clinkers (51 and 55 kJ/mol [37,38]). This higher activation energy of the AWLC material compared to conventional clinkers was attributed to the formation of structurally different hydration products, with a higher CSH mean chain length, low Ca/Si ratio, and higher crystallinity [18].

#### 3.2.2. FTIR and NMR Analysis

**Figure 4:**
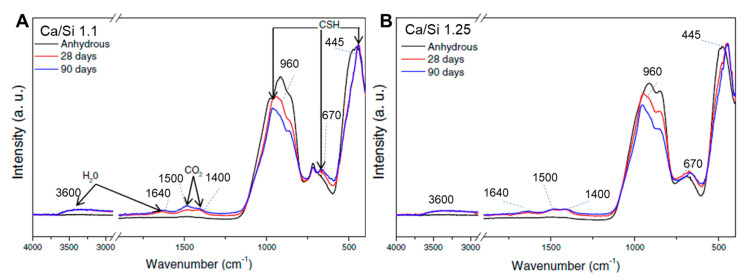
Comparison of the anhydrous AWLC FTIR spectra with a 28- and 90-day hydrated sample. The tests were performed on binders with a Ca/Si ratio of either (**A**) 1.1 or (**B**) 1.25. Adapted from [18].

**Figure 5:**
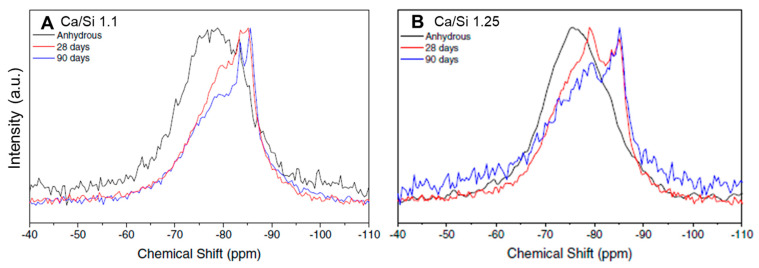
Comparison of the anhydrous AWLC ^29^SiMAS NMR spectra with a 28- and a 90-day hydrated sample. The tests were performed on binders with a Ca/Si ratio of either (**A**) 1.1 or (**B**) 1.25. Adapted from [18].

Santos et al. [18] performed FTIR and NMR analyses on anhydrous samples and compared them with 28- and 90-day water-hydrated pastes to assess the structural development upon hydration. Both binders were melted at 1550 °C and had a Ca/Si ratio of either 1 or 1.25. Figure 4 and Figure 5 depict the FTIR and NMR spectra, respectively.

Upon hydration, the FTIR spectra of both samples (Ca/Si of 1.1 and 1.25) revealed the development of a narrow band centered at −445 cm^−1^, indicating the formation of a more organized structure and a similarity between the hydration products [18]. All hydrated samples exhibited features at ~670 cm^−1^ and ~960 cm^−1^. The first peak is characteristic of the Si–O–Si bending mode related to CSH gels with a low Ca/Si ratio [18] and the peak at 960 cm^−1^ to the stretching vibrations of the Q^2^ tetrahedra [39]. In the range of 1400 to 1500 cm^−1^, the presence of an asymmetric stretching of CO_3_^2-^ is shown [40]. Finally, at 1640 cm^−1^ and 2800 to 3600 cm^−1^, two broad features can be assigned to the H–O–H bending vibration of molecular water and the stretching vibrations of O–H [40].

The evolution of the normalized ^29^Si MAS NMR spectra reveals that as the hydration progressed, the resonances moved to more negative chemical shifts, indicating an increase in the degree of polymerization achieved by the rearrangement of the least coordinated Q^n^ units (Q^0^ and Q^1^) [18]. Furthermore, the authors reported that the well-defined peaks of the hydrated sample at −79 and −85 ppm indicate the formation of a CSH structure, because these peaks are characteristic of end-chain Q^1^ groups (−79 ppm) and middle-chain Q^2^ groups (−85 ppm) with nonbridging Si-O-Si linkages. The presence of the Q^2^ component increases with age, reaching ~75% after 90 days of hydration, while Q^0^ groups disappear after this time of hydration [18].

#### 3.2.3. HR-TEM and XRD

**Figure 6:**
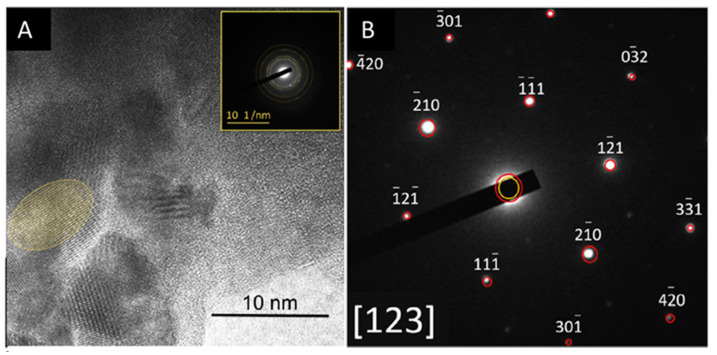
Selected area electron diffraction of TEM observations of a hydrated AWLC sample. Adapted from [41]. (**A**) HR-TEM image (**B**) experimental SAED pattern along the [123] zone axis.

**Figure 7:**
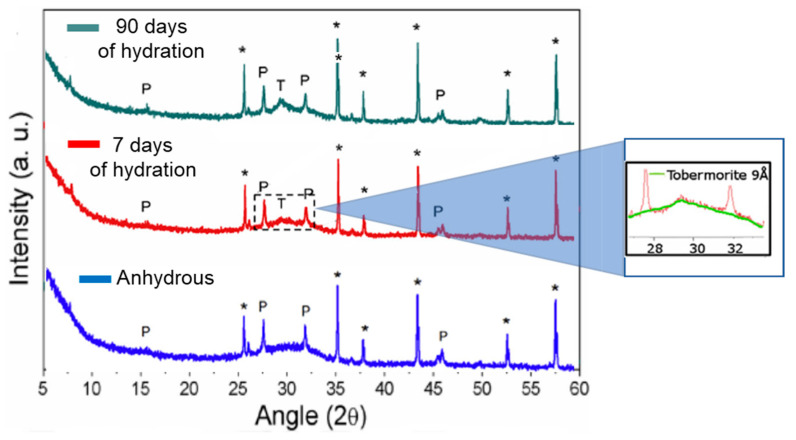
Comparison of Rietveld XRD analysis with a 7- and a 90-day hydrated AWLC sample. P—Pseudowollastonite; T—Tobermorite; *—internal standard Al_2_O_3_. Adapted from [41].

Paradiso et al. [41] performed selected area electron diffraction in HR-TEM observations of the hydrated product. It was observed that the CSH that formed upon hydration presented well-ordered nanocrystals with dimensions of the order of 10–20 nm and whose diffraction pattern is compatible with 9Å tobermorite (Figure 6). The presence of CSH and the tobermorite phase were also confirmed with Rietveld XRD analysis, which also showed that the proportion of 9Å tobermorite increased with the hydration time (Figure 7).

Computational simulation studies have previously shown that a low C/S ratio promotes a CSH with a more well-ordered lamellar structure that enhances the mechanical stiffness and hardness of the material [42]. The HR-TEM and Rietveld observations by Paradiso et al. show that within the binding phase of CSH, densely packed tobermorite 9Å nanocrystals formed in the AWLCs. Tobermorite is a layered structure composed of stacked Ca-O layers supported by silica tetrahedra, arranged according to the Dreierketten rule [1]. Depending on the degree of hydration, tobermorite can be categorized into three types, with a basal spacing of 9Å, 11Å, and 14Å [42]. The presence of tobermorite has also been documented during the hydration process of OPC [43]. Previous studies proposed mechanistic pathways for its formation, including the formation of amorphous and semi-crystalline CSH, followed by the growth of semi-crystalline tobermorite and, finally, the recrystallization of solid tobermorite [44]. The growth of this structure is facilitated by a mixture of heterogeneous nucleation and internal restructuring [45]. The ultra-confined interlayer of water within the tobermorite molecular structure influences the uniaxial tensile and compressive response of the structure [45,46]. In the case of the AWLCs, the observed tobermorite 9Å presented layers that were slightly inclined in the axial direction, and the structure did not contain any water molecules within its interlayer spaces. Previous studies reported that the tilting of the tetrahedra in the silicate chains and the shortening of the axial Ca-O distances allow for better dissipation of energy under compression, thereby improving the mechanical resistance of the material [46].

### 3.3. Relationship between the Tobermorite and Pseudowollastonite Content and the Mechanical Performance

Different melting temperatures were used to produce AWLC clinkers with different pseudowollastonite proportions [15,27]. Pseudowollastonite is a polymorphous wollastonite consisting of isolated trisilicate ring structures in which the calcium cation is ionically linked to oxygen atoms [47]; this crystal has a pseudohexagonal structure, whereas wollastonite has a triclinic crystal structure [48]. Wollastonite, which is characterized by its high thermal stability, low thermal expansion, and low thermal conductivity, has a chain structure and a density of 1.75 g/cm^3^ [49]. With an increase in temperature, wollastonite undergoes a reaction to form pseudowollastonite at 1125 °C [48]; this crystallization can be observed up to 1250 °C, increasing the density of the material to 1.98 g/cm^3^ [49]. The use of the pseudowollastonite phase has already been studied on low-calcium binders because of its carbonation capabilities, yielding CaCO_3_ and SiO_2_ as reaction products [50,51,52,53]. Plattenberger et al. [54] even proposed that the exposure of this phase to aqueous CO_2_ results in the formation of both CaCO_3_ and calcium silicate phases, which have been shown to be the more stable phases under low pH conditions.

**Figure 8:**
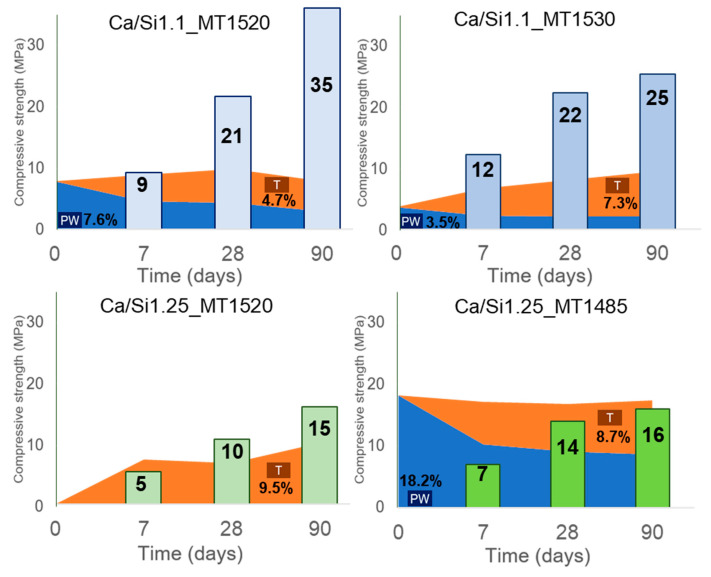
Correlation between the pseudowollastonite (PW, blue) and tobermorite (T, orange) content with the compressive strength of AWLC paste at 7, 28, and 90 days of hydration. The studied binders had a Ca/Si ratio of 1.1 (**top row**) or 1.25 (**bottom row**) and were produced with a melting temperature between 1485 and 1530 °C. Adapted from [15].

The samples tested in [15,27] were hydrated with water using a w/b ratio of 0.375 and submitted to microstructural and mechanical characterization [15]. Figure 8 resumes the obtained results. The results indicate that at early ages the compressive strength is more or less independent of the initial content of pseudowollastonite. However, at later ages (28 and 90 days), the samples with an initial pseudowollastonite content between 3.5% and 7.6% show better mechanical performance. In particular, samples with 7.6% pseudowollastonite show a significant evolution of the compressive strength, increasing from 8.5 MPa at 7 days to 34.5 MPa at 90 days. A decrease in the initial pseudowollastonite content and an increase in the tobermorite content for up to 90 days of curing time were also observed.

Therefore, this set of experiences shows that a higher pseudowollastonite content can be obtained by a lower melting temperature due to the equilibrium obtained in the liquid phase with the pseudowollastonite + liquid region of the CaO-SiO_2_ phase diagram, and the presence of small amounts of pseudowollastonite (up to 7.6%) in the AWLC may be beneficial in terms of mechanical performance. During hydration, the proportion of pseudowollastonite decreases, which may be a consequence of its carbonation process and at later ages could improve the compressive strength of the sample. Finally, it was possible to observe the presence of crystalline tobermorite during hydration, with its content increasing, at least, until 90 days of hydration.

### 3.4. Influence of Water/Binder Ratio, Granulometry, and Ca/Si Ratio on the Mechanical Performance

**Figure 9:**
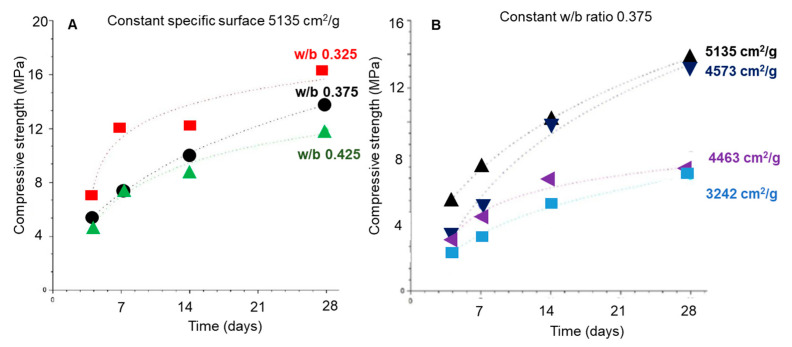
Compressive strength evolution as a function of the time of hydration for the AWLC paste with (**A**) different w/b ratios and (**B**) different specific surface areas. Adapted from [24].

The influence of the water/binder (w/b) ratio and the granulometry on the mechanical behavior is shown in Figure 9 [24]. It can be observed that both the w/b ratio and the particle fineness affect the strength of AWLC pastes, and the increase in the w/b ratio from 0.325 to 0.425 results in a significant decrease in the compressive strength of the material (Figure 9A). Furthermore, increasing its specific surface from 3242 to 5135 cm^2^/g results in a significant increase in the compressive strength. Nevertheless, Mendes et al. [30] reported that the effect of the particle fineness begins to fade at later ages (90 days) for binders with higher amorphous contents.

The compressive strength of the paste is influenced by both the specific surface area and the w/b ratio. These two conditions impact the exposed area of the particle and the distance between the binder particles, influencing the reactivity of the sample. When the specific surface area is high, indicating a larger exposed area, and the w/b ratio is low, indicating a shorter distance between particles [55,56], the conditions favor a higher reactivity of the sample. This increased reactivity promotes the production of more hydration products, specifically calcium silicate hydrate (CSH), resulting in higher compressive strength.

**Figure 10:**
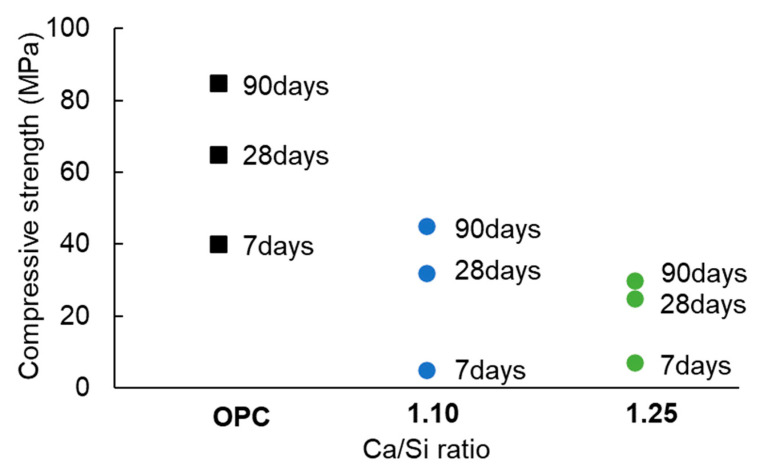
Comparison of the compressive strength of OPC (black squares) at 7, 28, and 90 days of hydration with the compressive strength of AWLC with different Ca/Si ratios. Blue dots indicate Ca/Si 1.1, and green dots represent Ca/Si 1.25. Adapted from [18].

The Influence of the Ca/Si ratio on the compressive strength of AWLC pastes was analyzed in [18]. The results are shown in Figure 10, using OPC pastes as reference material. It was observed that the samples with a Ca/Si ratio of 1.1 had better mechanical performances than the samples with a Ca/Si ratio of 1.25.

## 4. Alkaline Activation of the Binder

The use of alkaline activation to improve the compressive strength and performance of AWLC with a Ca/Si ratio of ~1.1 was studied by Santos et al. [16] and Mendes et al. [30]. The alkaline activators studied were Na_2_CO_3_, Na_2_SO_4_, CaSO_4_, and a mixture of Na_2_SiO_3_ and NaOH. The main observation of these works was that the AWLC activated with Na_2_SiO_3_ solution presented, by far, better performances. When Na_2_SiO_3_ solution is used as an activator, the mechanical strength of AWLC can overcome that of OPC. In this way, in this review, we refer only to activation with Na_2_SiO_3_.

**Figure 11:**
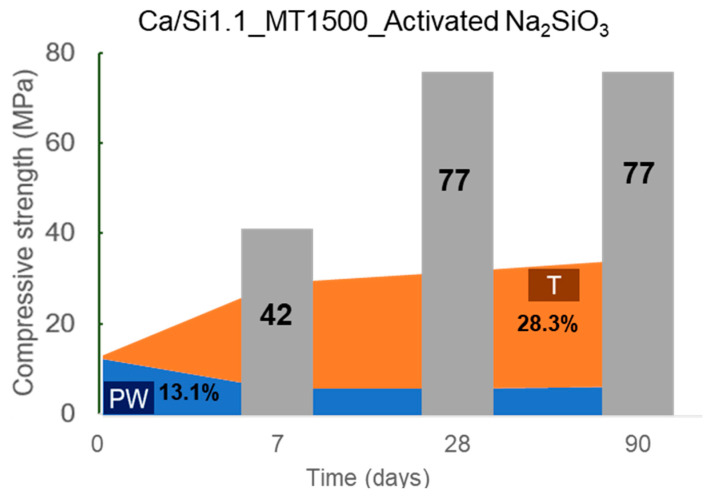
Evolution of pseudowollastonite (PW, blue) and tobermorite (T, orange) content, as well as its respective compressive strength at 7, 28, and 90 days of hydration, for pastes produced with AWLC. The studied binder was produced at 1500 °C and was alkali-activated with a Na_2_SiO_3_ solution. Based on [16].

Santos et al. [16] tested the compressive strength after 7, 28, and 90 days of hydration of pastes with a w/b of 0.375 activated with Na_2_SiO_3_. The compressive strength together with the evolution of the respective pseudowollastonite and tobermorite contents are shown in Figure 11. By comparing these results with those in Figure 8, for water hydrated pastes, it can be observed that the activation of the AWLC with Na_2_SiO_3_ promotes an increase in the compressive strength of up to 300%. Furthermore, the results indicate that the formation of tobermorite is related to the development of the mechanical properties of the pastes [14].

**Figure 12:**
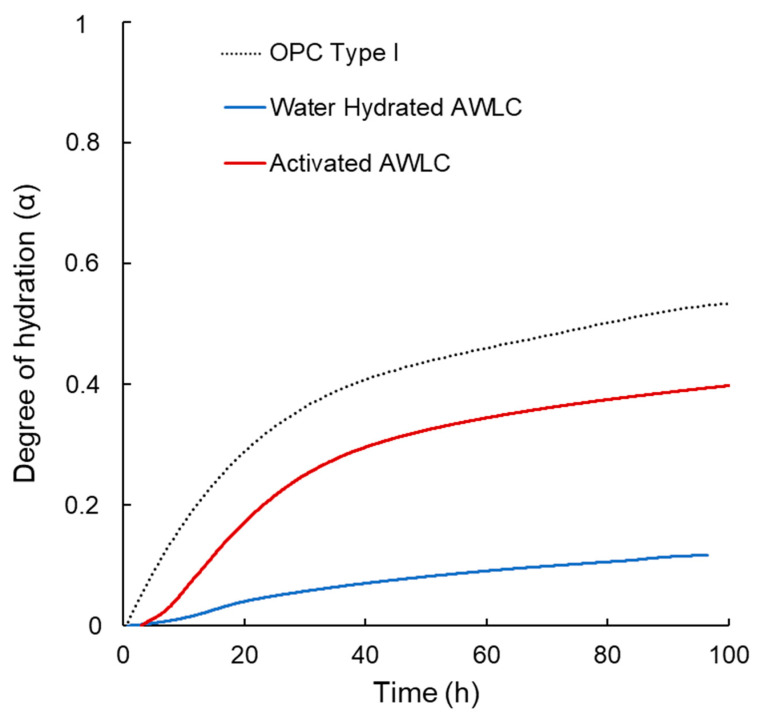
Hydration degree as a function of the curing time of type I OPC (black line), based on [57], and activated AWLC binder (red line), water-hydrated AWLC binder (blue line).

Since the degree of hydration (α) quantifies the extent of hydration of a binder over time, the experimental data from [17] were used to calculate the degree of hydration between water-hydrated AWLC and Na_2_SiO_3_-activated AWLC. We used the methodology proposed by Poole et al. [57], which calculated this parameter using the ratio of heat at each hydration time, H(t), over the total amount of heat available, H_max_, α = H(t)/H_max_. The results were compared with a typical type I OPC, as shown in Figure 12. The results in Figure 12 indicate that when the AWLC binder was hydrated with water, the degree of hydration remained below 0.1 for the first 100 h. However, by activating the material with Na_2_SiO_3_, a considerable increase in the degree of hydration was observed, indicating an increase in the hydration kinetics allowing for the formation of CSH/tobermorite structures, particularly at earlier ages, as observed using HR-TEM and XRD-Rietveld (Figure 6 and Figure 7).

**Figure 13:**
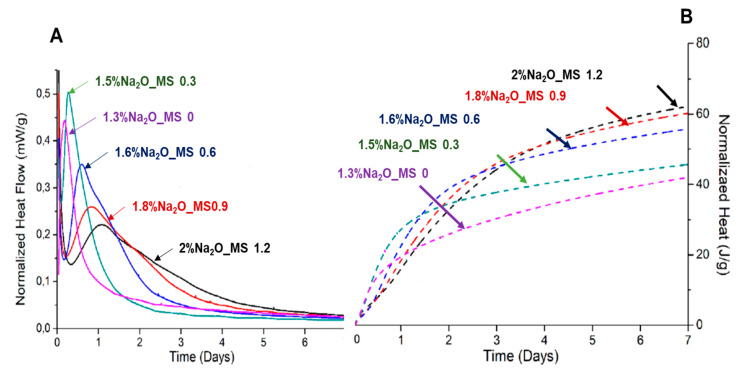
(**A**) Normalized heat flow and (**B**) normalized cumulative heat curves as a function of time of the hydration of AWLC samples with different amounts of Na_2_SiO_3_. Adapted from [30].

**Figure 14:**
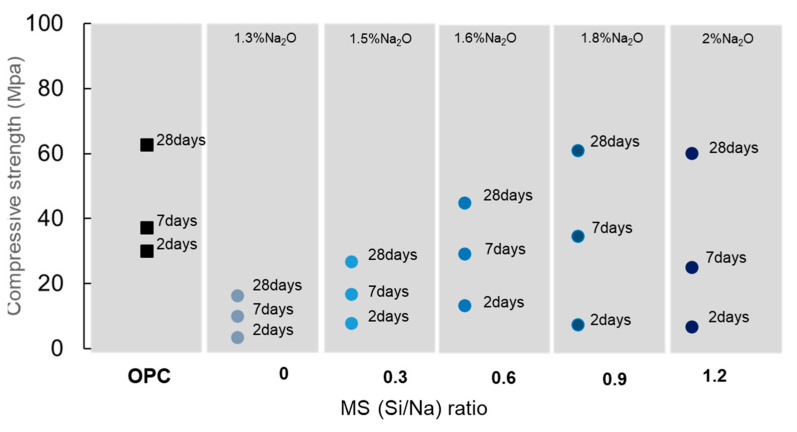
Compressive strength evolution over time for paste samples with different Na_2_SiO_3_ content. Adapted from [30].

To reduce the amount of Na_2_SiO_3_ and optimize the Na_2_O content in the hydrating solution, Mendes et al. [30] used a Na_2_O and Si/Na modulus of 1.2, followed by a successive reduction of 25%wt. of Na_2_SiO_3_ until a combination of just NaOH and water was reached (0% of Na_2_SiO_3_). The activation properties of each mixture were studied with calorimetry and compressive strength tests on pastes with a w/b ratio of 0.25. The calorimetric results and the compressive strength of each studied condition are shown in Figure 13 and Figure 14, respectively. The isothermal calorimetry analysis obtained by the authors showed a delay in the maximum hydration peak with the rise in Na_2_SiO_3_ on the activator. However, the increase in the Na_2_SiO_3_ content also promoted a more controlled kinetic after the peak; consequently, these samples released more heat after 7 days of hydration. As a result, after 7 days of hydration, the amount of heat released increased with the increase in Na_2_SiO_3_ concentration. The compressive strength results on pastes activated with the studied solutions showed that the samples with a higher heat released originated higher compressive strength results at later ages. Presently, there are no published studies on the activation mechanisms of Na_2_SiO_3_. 

Presently, ongoing studies are being conducted to explore the chemical factors that influence the activation of the binder.

## 5. Correlation between Bonded Water and Compressive Strength

Thermogravimetric analysis was used to calculate the amount of bonded water (BW) on the hydrated phases by measuring the weight loss of each sample in the temperature range of 110 °C to 500 °C [15,18,30]. Considering that the strength development of the samples is directly related to their hydration process, it is possible to correlate the compressive strength with the chemical BW content. Furthermore, assuming the model of Richardson and Qomi [43], a relationship between the bonded water and the amount of CSH can be established:

$$\frac{H_{2}O}{Si}=\frac{19\frac{Ca}{Si}-7}{17}.$$

**Figure 15:**
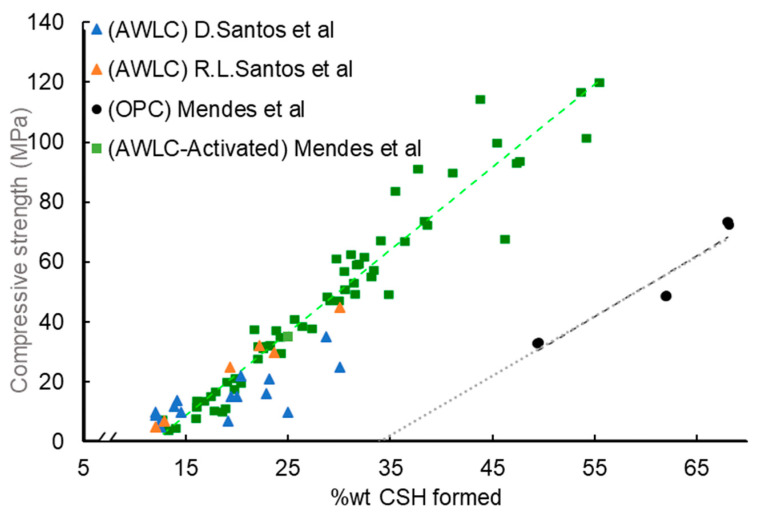
Plot of weight percentage of CSH formed versus compressive strength for water-hydrated OPC pastes and AWLC pastes hydrated with either water or Na_2_SiO_3_ solution. Adapted from [15,18,30]. The green trend line represents the relationship between the percentage of CSH formed and the compressive strength of AWLC pastes, while the black trend line represents the same relationship for OPC.

Using these data, it is possible to compare the percentage of CSH on the sample with its compressive strength, as shown in Figure 15. It can be observed that AWLCs require a lower amount of CSH to obtain compressive strength values similar to OPC. This may be due to the presence of hydration products with a lower Ca/Si ratio, which allows the formation of a CSH structure with better mechanical properties [42]. Moreover, the linear correlation between CSH formed and the respective compressive strength on all experimental pastes, hydrated with water or with an alkaline solution, suggests that the resulting hydration product is similar in all cases.

Finally, Table 2 summarizes the main characteristics of the AWLC binders compared with OPC. Other physicochemical characteristics of AWLCs compared with alite, α-belite, rankinite, α-wollastonite, mayenite, krotite, grossite, and gehlenite are presented and discussed in detail in Freitas et al. [33]. 

## 6. Conclusions

The development, optimization, and use of low-calcium amorphous hydraulic binders with Ca/Si ≈ 1 as an alternative to Portland cement-based materials aiming to reduce the carbon footprint associated with its production have made relevant progress since 2015. In summary, the main achievements are as follows:

(a) To obtain a binder with good mechanical performance, the raw materials should be heated to at least 1500 °C, and the quenching should be performed preferentially in water;
(b) With the increase in the calcium content of the raw material, with C/S between 0.8 and 1.25, an increase in the Q^0^ structures was observed, reaching a maximum value at a Ca/Si ratio of 1.1. Moreover, pastes prepared with this ratio showed an increase in compressive strength;
(c) The tests performed on the hydrated product revealed that the only products formed during the hydration of this AWLC were an amorphous CSH with a Ca/Si ratio of 1.1, an MCL of 5, and a crystalline tobermorite 9 Å phase. Furthermore, no portlandite was identified;
(d) With the increase in hydration time, a reduction in pseudowollastonite and an increase in tobermorite were observed. On water-hydrated pastes, the optimum content of pseudowollastonite on the anhydrous binder was ~7.6%;
(e) The degree of hydration rate of the AWLCs binder was established and compared with type I OPC, and the results show that even though the hydration rate of the binder was lower than that of OPC, by activating the material a significant enhancement in the degree of hydration was observed, suggesting a potential improvement in its performance;
(f) Competitive strength on pastes was only obtained when the binder was hydrated with a Na_2_SiO_3_ solution with at least 1.8 Na_2_O%wt. content. When activated, the pseudowollastonite content does not seem to be relevant to the performance of the binder. An important parameter observed was that the AWLC binder exhibited a much lower heat release than traditional type I OPCs, even when activated;
(g) Finally, a correlation between bonded water and the formation of CSH chains with compressive strength was established by different authors.

In this work, the studies performed for the production and characterization of AWLC binders were reviewed. The main results showed that unlike OPC, which predominantly contains crystalline phases (e.g., alite and belite), AWLC binder has an amorphous structure that allows for its chemical hydration. When hydrated with the proper alkaline activator the hydration product exhibits a compressive strength that can overcome those of OPC. In addition, the reduction of the calcium content in the AWLC binders leads to a significant decrease in CO_2_ emissions associated with its production process. However, to fully understand the potential of AWLC binders, further investigations are necessary. The production of the material on larger scales, as well as its testing in mortars and concrete, are crucial to evaluate its performance under real-world conditions. Additionally, a deeper understanding of the hydration reactions of the AWLC binder, particularly the role and influence of the activator, should be carried out in the near future.

## Figures and Tables

**Table 1:** Deconvolutions results of the NMR spectra. Adapted from [26].

	Ca/Si Ratio	0.8	1	1.1	1.25
Q^n^ Area (%)					
Q^0^		18.1	18.4	28.1	21.5
Q^1^		26.8	37.5	40.2	50.1
Q^2^		34.7	30.4	27.6	26.8
Q^3^		20.4	13.6	4.1	2.6

**Table 2:** Comparative summary of the main features of the AWLC binder with OPC [11,17,26,30,42].

	OPC	AWLC
Production method	The raw mix is fed into a kiln and fired to a temperature of 1400–1450 °C	Melting raw mix at ~1500 °C, followed by a fast quenching
Process-related CO_2_ (kg/ton)	535	340
The density of the material (g/cm^3^)	3.1	2.94 ± 0.05
Main phases of the binder	Alite (50–70%) Belite (15–30%) C_3_A and C_4_AF (<20%)	Amorphous (>90%) Pseudowollastonite (<10%)
Activation energy (kJ/mol)	51–55	82–85
Cumulative heat after 72 h (J/g)	250	53 when activated with Na_2_SiO_3_ solution with 1.8%wt. Na_2_O content
Main hydration products	CSH and portlandite	CSH and tobermorite 9 Å
Ca/Si ratio of CSH	~1.7	~1.1
MCL	~2.4	~5
Paste compressive strength—2 days (MPa)	33	11 when activated with Na_2_SiO_3_ solution with 1.8%wt. Na_2_O content
Paste compressive strength—28 days (MPa)	65	63 when activated with Na_2_SiO_3_ solution with 1.8%wt. Na_2_O content
